# Ophthalmia

**Published:** 1825-04-01

**Authors:** 


### OPHTHALMIA.

1. *Observations on the History and Treatment of the Ophthalmia accompanying the secondary forms of Lues Venerea. Illustrated by Cases.* By THOMAS HEWSON, A.B. Member of the Royal College of Surgeons in Ireland; Professor of Materia Medica, &c. and Surgeon to the Meath Hospital and County of Dublin Infirmary, &c. &c. &c. Octavo, pp. 117. With a coloured plate, containing six figures. London, 1824.

2. *Practical Remarks. Part* I. *On Acute and Chronic Ophthalmia. Part* II. *On Remittent Fever, viz. Simple and Complicated.* By THOMAS O'HALLORAN, M.D. Author of Treatises on the Barcelona and Andalusian Yellow Fever Epidemics. Octavo, pp. 148. London, 1824.

IT is very probable, as Mr. Hewson observes, that venereal ophthalmia is coeval with the other secondary symptoms of syphilis, among which it holds a distinguished place. It is therefore remarkable that more has not been written on the subject, considering the importance of the organ affected. None of the earlier, and but few of the later authors, according to Mr. Hewson, "have manifested the most distant notion of the true seat or symptoms of this affection"—even Hunter himself entertained doubts of its existence. The descriptions, says Mr. Hewson, of Howard and Ben. Bell are "devoid of every degree of accuracy and truth." Mr. Pearson also, in a letter to Mr. Briggs, the translator of Scarpa, observes that "there are no specific characters by which diseases of the eyes or eyelids, produced by the action of the venereal virus, can be distinguished from those excited by other causes." The merit of being the first author who made this ophthalmia an object of particular attention, is due to the late Mr. Saunders, who took a correct view of its distinguishing symptoms. His untimely death left this and most of his investigations unfinished. Although this ophthalmia has attracted pretty general attention since Mr. Saunders' time, Mr. Hewson avers that "it is not yet distinguished with sufficient accuracy from the common kinds of ophthalmia—nor is the proper treatment observed in these respective states of disease." Mistakes of this kind, he remarks, bring injury to the patient and discredit on the practitioner. On this account he thinks that a short and distinct practical view of the syphilitic ophthalmia, divested of those speculative doctrines in which the whole subject of lues venerea has lately been involved, may be useful to the junior members of the profession, "for whose instruction it was chiefly undertaken."

When syphilis attacks the internal structures of the eye, a peculiar ophthalmia is the consequence, which has lately been termed venereal iritis—a term which our author thinks objectionable, as limiting the disease to the iris. He therefore prefers the older denomination *syphilitic ophthalmia*. He divides the progress of its symptoms into two stages. In the *first*, they are all of a temporary nature, and effectually curable—in the *second*, they are, in a great degree, beyond the reach of relief, and more or less permanently injure the eye in structure and function. This disease attacks one eye first, and after having gone through its various stages, seizes on the other. It is very seldom found to exist in both eyes at the same time, unless neglected or unskilfully treated. But the eyes are sometimes alternately affected, the disease subsiding in one, and becoming aggravated in the other.

1. *First stage*. As our diagnosis depends on accuracy and fidelity of description, it will be necessary to be somewhat minute in this part of the subject. We shall open the description in Mr. Hewson's own words.

"It usually begins with some uneasy sensations about the ball of the eye; sometimes these are so slight as not to be complained of: there is generally a flow of tears; the admission of light gives pain; and objects appear as if seen through a mist. Commonly, however, these symptoms at first engage so little of the attention of the patient, that he neglects applying for advice until urged to it by the increasing dimness in his sight. It is therefore unusual to meet a case in which the disease has not been of some duration, and made more or less progress.

"The following appearances and symptoms, as they affect the different parts composing the ball of the eye, will, in a majority of cases, be observed.

"The patient keeps his eye half-closed, and avoids, as much as possible, exposure to the light, which always excites pain and flow of tears: from the same cause the eye rolls, and is so unsteady, whilst an examination is making of it, that it is often difficult to get a satisfactory view of it.

"There appears always more or less external inflammation; and if we attentively examine the enlarged vessels, we shall, in general, be able to distinguish two tissues of them; one superficial, which belongs to the conjunctiva; and the other more deep-seated, connected with the substance of the sclerotic: in colour, they are sometimes of a florid, but oftener of a purple or venous hue. That part of the conjunctiva which lines the eye-lids is not usually under much irritation; the portion which covers the eye-ball is a little more inflamed; sometimes it is here a little œdematous, or a small vesicle or two may be observed on it; but it never enters into the thickened, fungous structure, nor is there any secretion of purulent mucus from its surface; both of which are so often the effects of the severer forms of external ophthalmia.

"A great part, however, of the redness of the eye-ball is produced by vessels passing through the sclerotic, which becoming more numerous and frequent in their inosculations as they advance forward, and terminating abruptly, nearly at the space of a line from the cornea, about the situation of the ciliary ligament, cause in this part the peculiar appearance of almost a distinct red circle. Neither at this, or the more advanced periods of the disease, do these enlarged vessels pass over the surface of the cornea; nor have I ever observed any speck or ulcer forming on it: in a few instances there has been an appearance like the arcus senilis.

"There is generally a pretty abundant flow of tears, the ordinary motions of the eye and eye-lids; the effort to look attentively at any object, or any pressure on the eye-ball, create pain. These symptoms may surprise an inexperienced observer, as not being sufficient to account for the great defect of vision of which the patient complains; but the principal causes of this will be found to exist in the state of the aqueous humour and pupil, to which the attention must be particularly directed. This humour is always more or less clouded by an opaque fluid, which is generally seen floating in the anterior chamber. This prevents a clear view of the iris and pupil, and causes an appearance as if there was an opacity in the cornea. As to its nature, this opaque fluid would appear to be lymph, secreted by the vessels of the inflamed iris. It varies in quantity; it is sometimes so abundant as to render the greater part of the iris invisible; and, intercepting all passage of the rays of light, for a time entirely deprives the patient of vision. In some cases it scarcely disturbs the transparency of the aqueous humour; in others we see it condensed and collected into a mass, and attached to some part of the iris, generally near the pupil. Whilst in this state, in the more advanced stages of the disease, vessels shoot into it, and it becomes organized. It would seem to be specifically heavier than the aqueous humour, for it changes situation according to the position of the head. Thus, after the patient has lain some time in bed, it may be seen on either side of the anterior chamber; but when he is for any time in the erect posture, it accumulates chiefly about the lower part of the chamber and inferior margin of the pupil; also, as more or less of it always lies within the space of the pupil, it produces here an appearance like commencing cataract." 7.

In a few instances our author has seen blood instead of lymph effused into the anterior chamber, but not attended with any bad effects.

The appearances about the pupil vary in different cases—

being in no two instances exactly similar. In general it is within the middle state of expansion, and remains nearly immoveable in every variation of light. As the disease advances, the pupil contracts, and soon begins to lose its circular form. Some part of it, however, usually preserves its natural form, contracting a little under light, and dilating under belladonna. In many cases, one or more small tubercles present themselves about the pupil, at this period—often appearing on some part of the surface of the iris, which undergoes some remarkable changes as to colour, often assuming a yellowish or amber tinge—sometimes with a reddish circle about the ciliary ligament. As the disease advances, vision grows more imperfect, and rather severe darting pains are often felt at the bottom, and round the external parts of the orbit. In many instances, however, no other unpleasant sensation is perceived beyond that of intolerance of light.

In this state, or nearly so, the symptoms may remain from six to eighteen days—being seldom protracted beyond the twentieth, without symptoms of the second stages presenting themselves. It is to be borne in mind that, at this period, while the pupil is contracted, and its motions impeded, adhesions may form between it and the capsule of the lens, without being immediately perceptible. If this has occurred, the disease has already entered into the second stage, which is now to be described.

"2. *Second Stage.*—In addition to the preceding symptoms, we will have to notice, in the second stage, an increased contraction of the pupil, and that it becomes more irregular, puckered, and inverted; and often a black fringe appears round its border as if from a detachment of portions of the nigrum pigmentum. We will, also, generally see one or more angular points proceeding from it towards the capsule of the lens, and these mark where adhesions have formed between it and the capsule. For a greater or less extent round these attachments, the latter membrane gradually grows opaque. The whole surface of the iris is often observed to become conical, and to diminish considerably the limits of the anterior chamber. About this time, also, in a few instances, enlarged vessels may be distinguished, ramifying superficially over it.

Likewise, we will have, in many cases, to attend to a very singular, and, I might add, characteristic symptom, to which I have already alluded, namely, the formation of one or more small tubercles on some part of the iris. When these occur, there is always more pain and tenderness felt in the eye-ball than usual: they commonly present themselves at or near the surface of the iris, between the pupil and its ciliary attachment; they are found from the size of a large pin's head to that of a small split pea; sometimes there appears but a solitary one, at others, we see two or more of them unconnected; but, in some cases, a number are clustered together, and project into the pupillary space, so as nearly to fill it up, or protrude forward into the anterior chamber: at one time they hang pendulous, at another, they are attached by a broad base; when small, they are of a dark red colour; but when large and prominent, they are more or less white at the apex, while, about the base, the redness continues. By closely examining them in this latter state, we shall distinctly observe that the inflamed superficial membrane of the iris is reflected over them, and forms their anterior covering; and thus, when these tubercles are small, their covering retains the redness caused by its state of inflammation; but when they are larger and more pointed, it becomes transparent about the apex, where, as happens in a common pustule, their whitish contents are visible." 14.

After the disappearance of these tubercles, we may often discover a fissure, or cicatrix in that part of the iris where they have been situated. Our author considers them of a pustular or purulent nature, and has never found them attendant on any other kind of inflammation or morbid action. He, therefore, looks upon them as characteristic of venereal ophthalmia.

"During the active stages of the disease, the pupil still shews a tendency to contract, until, at length, it scarcely exceeds, in diameter, a large pin's head. The opacity likewise spreads, and becomes more dense in the capsule of the lens; and, finally, nearly all power of vision is suspended." 16.

In this state of things, there is every probability that the deeper seated coats, the choroid and retina, are more or less involved in the disease. In the syphilitic ophthalmia, indeed, defective vision and intolerance of light are amongst the earliest symptoms complained of, occurring before any decided signs of disease appear about the iris and pupil, and continuing unabated, or rather increasing, while any degree of the morbid action is going on.

About this period—sometimes earlier, if no efforts are made for the relief of the complaint, the other eye may be attacked, and the symptoms suspended in the first—and thus alternating till the disease has produced its full effects in both. In some time after the symptoms have completed their course, they generally begin gradually to assume a more indolent and chronic form, and the pain and irritation finally to subside.

After the disease has been sometime established, and where an irregular and inefficient treatment has been pursued, an abscess sometimes forms in the deep seated parts, and generally terminates in the destruction of the organ. The first indication of this, is usually an œdematous swelling on the fore part, and on one side of the eye-ball, immediately behind the ciliary attachment of the iris. The abscess makes its way, both externally and into the anterior chamber, where it discharges itself in flakes that do not mix with the aqueous humour, as happens in common hypopion. Both iris and cornea are quickly destroyed by sloughing and ulceration, the aqueous humour, lens, and part of the vitreous humour, are evacuated—the sclerotic contracts about the vacant space, and the anterior chamber is finally obliterated.

*Constitutional Symptoms.* It is only when syphilis has attained its secondary stages, and more or less contaminated the system, that the eye becomes susceptible of the venereal ophthalmia.

A half-cured chancre, or bubo, may be present with it, but ome constitutional taint is necessary to its production. Some facts have led our author to believe that the violence of the ophthalmia is in proportion to the disturbance and strength of the constitutional symptoms, and *vice versâ*. He has also observed that, in those cases where no mercury has been used in the primary stage, or previous to the occurrence of the ophthalmia, the latter has appeared in its most severe and exasperated forms, and *vice versâ*. Thus, nurses who receive the infection from pocky children, and women who receive it from their husbands, often allow the disease to run on to its second stage unsuspected, and in them the venereal ophthalmia, when it does occur, is generally of a bad kind. "On the other hand, the patients with whom the ophthalmic symptoms have manifested most mildness and indolence (who are by far the majority) have been those whose previous treatment had been conducted on what is called the alterative plan—by pills, and without confinement."

Some species of eruption most commonly attends the syphilitic ophthalmia, and is generally of the papular or scaly kind. It is not, however, necessarily connected with eruptive symptoms—it may be associated with any general symptom—or it may exist alone. When the characters of the constitutional symptoms are unequivocal, their concurrence will be decisive of the nature of the ophthalmia—but when they are faint or obscure, other circumstances are to be looked into, as the state of health—some previous syphilitic affection, &c.

"If, in conjunction with some suspicious appearance about the skin, we observe him pale and emaciated, that his health and strength have been for some time on the decline, that he is less equal to his usual bodily exertions, that he complains of pains about his limbs and joints, particularly at night, together with nocturnal perspirations; that at some recent period, generally within the twelvemonth, he contracted venereal symptoms, in the treatment of which there appears something faulty or objectionable; we have strong grounds for concluding the case to be syphilitic.

"If the skin is free from any symptom to which we can attach suspicion, we are next to examine into the state of the throat, which is very frequently affected at the same time with the eye. We shall here often find one or more ulcers, or a greater or less degree of excoriation, or irritation, either actually present with, or immediately preceding, the ophthalmia; and, in some instances, the transition of the disease from the throat to the eye has been remarkably rapid, a day or two only intervening between its disappearance from one and its seizing on the other. As the symptoms about the throat are often of an extremely dubious character, we can seldom solely rest our judgment on them; but must, as has been observed above, take our view of the case from an enquiry into the other circumstances attending it." 28.

Besides the above, there may be discovered, on different parts of the body, morbid cicatrices, blotches, or ulcers—there may be articular or periosteal pains or swellings, &c. But, in not a few instances, the ophthalmia may be solitary. In women, we shall often find that, in addition to the ophthalmia, the only circumstance leading to a suspicion of constitutional taint, is their having had repeated abortions, or still-born children.

*Exciting Causes.* These are such as are found to be influential in exciting the venereal morbid actions in other parts of the body—particularly exposure to wet and cold. Our author does not concur in the opinion of Mr. Travers, that the constitutional use of mercury predisposes to the disease. "I have, says Mr. Hewson, cæteris paribus, as frequently met the ophthalmia where no mercury had been previously used, as any other secondary symptom of lues venerea; and, indeed, some facts have induced me to believe, that the constitutional operation of mercury rather diminishes than increases the susceptibility to the former."

*Diagnosis.* It is only the milder and more chronic forms of internal *idiopathic* inflammation that have any similarity to those of a *syphilitic* origin. But notwithstanding their agreement in some respects, idiopathic will be found to differ in its effects from the syphilitic ophthalmia, in many remarkable circumstances.

"1st. It does not so very quickly disorganise the structure, or disturb the functions of parts it attacks; and it is often for months' duration, sometimes subsiding, or almost entirely disappearing; and again, after certain intervals, returning, without leaving any permanent injury.

"2d. We shall rarely observe (indeed I have never met such a case) that it produces the same kind of pustular tubercles on the iris, and about the pupil, which have been mentioned as frequently appearing in the venereal ophthalmia.

"3d. After it has subsisted for a long time, it frequently causes organic changes in the iris, choroid, retina, and in the vitreous humour, and terminates in amaurosis, sometimes with an adherent and contracted pupil; in others this is dilated, and in these cases, it is not unusual to observe, in the same individual, one pupil amaurotically expanded, and the other closed.

"Finally, mercury alone is not so active, or so certain in subduing idiopathic inflammation of these parts, as some writers suppose; whilst the means necessary to resort to for this purpose, and which most frequently succeed, are totally useless in the venereal ophthalmia." 35.

But although many other points of difference will readily occur to the experienced practitioner, still no reliance should be placed on lines of distinction drawn from topical appearances alone. In all cases, the state of the patient's general health, both previous and subsequent to the occurrence of ophthalmia, should be closely investigated.

Passing over capsular inflammation, rheumatic and arthritic ophthalmia, we come to—"*ophthalmic symptoms caused by mercury.*" Our author observes that, between this and syphilitic ophthalmia it is equally necessary and difficult to draw clear lines of distinction. Mr. Travers appears to our author to identify the two sources—averring "that it is impossible to say to which of the two agents the effects belong which manifest themselves at a period subsequent to their successive introduction into the system." To Mr. Hewson this statement does not appear to be borne out by facts.

"It is not because a majority of the cases of iritis met with 'are consequent on the use of mercury,' nor yet because they sometimes occur 'while the system is charged with mercury,' that we are authorized in concluding one to be the specific effect of the other; for we might with equal reason assign mercury as the cause of the greater number of constitutional symptoms generally acknowledged to be syphilitic, the cases in which they appear being commonly found under similar circumstances. A more obvious, and, in my mind, a truer source of the cases in question, may generally be traced to a defective and inadequate mode of administering mercury; in consequence of which, besides other effects, it fails to exert its anti-syphilitic properties, or to eradicate the disease from the constitution; and, during its exhibition in this way, and while the system is yet under its influence, we frequently find syphilitic symptoms appearing in succession, or retiring from one place, and breaking out in another, and often committing some of their severest ravages. This is no uncommon result of what is termed the alterative plan of treatment, which is confined to the administration of pills, and in which the patient is placed under no salutary restrictions; and ophthalmia, like other syphilitic symptoms, (as will appear from a perusal of many of the cases subsequently detailed,) is no very unfrequent consequence of it. A minute investigation, however, into an extensive variety of cases, which came under my observation while acting for some years as surgeon in the Lock Hospital of this city, (perhaps at the time the largest establishment of the kind in Europe,) not alone impressed me with the opinion that iritis occurring under the circumstances above stated is, with very few exceptions connected with a syphilitic taint of the constitution, but also that the ophthalmic symptoms usually associated with the 'constitutional use' of mercury are of a very distinct and different nature from the disease in question. Indeed, my experience leads me to look on primary iritis as a very rare effect of the constitutional use of mercury. On the contrary, I have observed the external structures remarkably subject to be morbidly affected by it, and this without its manifesting any tendency of the same kind elsewhere." 48.

In many instances, our author has thought that mercury created a susceptibility to conjunctival inflammation, or aggravated it where present. By this he would not be understood, however, as maintaining that it is often the consequence of a short and temporary use of mercury—but that he has frequently found it resulting from a negligent and protracted exhibition of it, "particularly where it was not calculated to act as a remedy."

Mr. Hewson avows that, in those complicated and anomalous cases, which are but imperfectly known to us, under the denomination of *pseudo-syphilis*, where there are "grounds for suspecting that the syphilitic and mercurial poisons are acting on the system at the same time," we shall frequently find it extremely difficult, if not impossible, to form any diagnosis; but, at the same time, diagnosis is here unnecessary—"for we must always treat ophthalmic symptoms occurring under these circumstances, and of this nature, as if they were of a genuine and unmixed syphilitic origin." "For if we here wait the slow effects of any other treatment than that with mercury, these delicate organs will, with certainty, sustain irreparable injury." 55.

*Prognosis.* On this head little need be said. The symptoms of the first stage are easily and speedily subdued by proper treatment; but many of those attending the second, such as an adherent and contracted pupil, and opaque capsule, remain, to a certain degree, permanent and irremediable.

*Treatment.* " It is not a little remarkable, that there are no symptoms of lues venerea, whether primary or secondary, or wherever situated, which yield with more facility or certainty to the constitutional action of mercury, than those which form our present subject—a circumstance peculiarly fortunate, considering the delicate structure of the organ concerned, as well as the rapidity with which many of the most lasting effects of the disease are produced. We have, therefore, little more to do than consider of the most active and efficient mode of bringing the patient under the influence of this medicine." 57.

The oxymurias hydrargyri is a favourite remedy on the Continent, and even with some practitioners in these Islands. But it is objectionable, inasmuch as it must be introduced slowly and in small doses. It moreover acts usually in the alterative way, which is inadequate to a cure in this complaint. The mode of administering mercury now generally adopted in Dublin consists in exhibiting a pill containing two or three grains of calomel, and half, or a quarter of a grain of opium, night and morning, or sometimes oftener—varying these proportions according to their effects or the urgency of the symptoms. The opium is useful in allaying pain or irritation, and in preventing the calomel from running off by the bowels. In proportion as the general system becomes affected with the mercury, we shall observe a gradual amendment in the eye. The pain and irritation first cease—the redness of the eye-ball, the intolerantia lucis, the lachrymation subside—the transparency of the aqueous humour is restored—the pupil assumes its natural actions—and all morbid depositions and appearances are removed. Finally, though sometimes slowly, vision returns to its natural state. This, of course, presupposes that the treatment has been taken up under favourable circumstances, and during the first stage of the disease, otherwise our success will be more slow and less complete.

"If, after pursuing the preceding treatment for six or eight days, the mercurial influence does not manifest itself, or the symptoms with which the eye is affected do not sensibly yield, frictions may be combined with the pills, until ptyalism is fully established; from the first appearance of which, we shall usually find that the symptoms take a favourable turn. But if, after this sets in, no decided amendment follows, we may reasonably suspect the case not to be of a genuine venereal kind. Though frictions are, with a few exceptions, to be preferred to all other modes of using mercury in the treatment of syphilitic symptoms, in those affecting the eye they are not sufficiently active; nor would it be prudent, in the first instance, to join them with the pills, because these latter will seldom disappoint us; and by attempting to introduce mercury too rapidly into the system, we often defeat our purpose of exciting its proper constitutional action, and render this afterwards more difficult of accomplishment." 60.

In the treatment of this ophthalmia confinement is indispensible. Blood-letting and blistering are considered by our author as perfectly useless, and the same with purgatives. They ought therefore, in his opinion, to be avoided. Mr. Hewson, however, must know that venesection is a powerful promoter of the constitutional action of mercury, and may therefore be a proper adjuvant on particular occasions. No advantage is to be expected from collyria of any description. Some ease is experienced from fomentations of warm decoction of poppy-heads, in which some of the watery extract of opium may be dissolved. Belladonna is often applied, but Mr. Hewson thinks that it is unnecessary, or even improper, till all active symptoms have subsided.

"When all morbid action has been removed from the eye, and its functions have been restored, as far as its state will admit of, we are not to stop here in the exhibition of mercury, but must persevere in its use as long as we have any suspicion of the existence of a venereal taint: for it should be kept in view, that the quantity required for the relief of the ophthalmia, falls far short of what is necessary for the complete eradication of the disease from the system. In those cases where the syphilitic poison has heen allowed to lurk in the constitution, the ophthalmic symptoms, though in a degree subdued, have appeared to proceed on almost imperceptibly, and a gradual and nearly total failure of vision has ensued, which can only be restored by a full and efficient course of mercury." 63.

In conclusion, Mr. Hewson differs from Mr. Travers, who considers "that all forms of iritis, whether primary or secondary, simple or specific, require the constitutional use of mercury for their cure, without exception." Mr. H. thinks that there are many cases of idiopathic iritis where mercury, except as a purgative or corrector of depraved secretions, is unnecessary, as, for instance, where the source of the ophthalmia is disordered condition of the digestive organs—or of the brain. Between these authors we shall not attempt to stand umpire. We have set both their opinions before our readers.

We have now gone through the didactic portion of Mr. Hewson's volume, and given a very copious analysis of its contents. The narrative portion, occupying nearly half of the publication, and containing numerous instructive cases, we recommend to the careful attention of all those interested in the treatment of syphilitic ophthalmia. We have also to return thanks to the author for the instruction which we have derived from a perusal of the results of his practical observations. The plate, containing six coloured figures, is very well calculated to aid the verbal descriptions, and convey clear ideas of the complaint to the reader.

We now come to Dr. O'Halloran's practical remarks on acute and chronic ophthalmia, &c. derived chiefly from military practice, especially in the 64th regiment—a corps in which he has served, in various climates, from the year 1813 till very lately. Our author remarks in his preface, that he pursued the antiphlogistic plan of treatment in ophthalmia, for a period of twelve years, "with a success far short of what he was taught to expect from depletion in diseases of a purely inflammatory character." In fact, in some cases, it was observed that, as soon as the patients recovered from long-continued syncope, "the turgidness of the blood-vessels of the eye returned, and the cases were more troublesome, and the cures more protracted, than where blood had been more moderately abstracted." Experience of twelve years also taught our author "that ophthalmia was aggravated by low diet, total abstinence from wine, depletion, and confinement to bed." It was observed that those practitioners who bled most copiously were least successful, and, finally, that the exclusion of light and air was highly injurious. Reflecting on these things, our author concluded he was not in the right track, and, at length, struck into that which is here laid before the public.

The unusual prevalence of ophthalmia in the 64th regiment, at Gibraltar, during the two last years, afforded the medical officers but too extensive a field for observation. It is a curious and a melancholy fact, that ophthalmia is more unmanageable in military than in civil life. "The remedies which produce effect with almost positive certainty in one, and that in a short time, are wholly inefficacious in the other—a circumstance that, with the augmented malignancy under which the disease appears among the troops in hot countries and crowded quarters, has excited the astonishment of many whose walk has been confined to the dispersed inhabitants of cold or temperate climates." It is also astonishing, that in a garrison—containing several regiments, one corps will have an ophthalmic list of from 30 to 50, whilst the others are, in a manner, exempt.

I. *Acute Ophthalmia.* Dr. O'Halloran describes three varieties of acute ophthalmia, as it appeared at Gibraltar, in the 64th regiment.

1. The *first* exhibited redness of the conjunctiva and lining membranes of the eye-lids—inconsiderable lachrymation—slight turgescence of vessels—sensation of sand in the eyes—vision little, if at all, affected. Such were the symptoms of the first variety, and it frequently yielded to simple treatment, in a few days.

2. The *second* variety was more complicated. The attack is sudden—there is an increase of lachrymation—a villous flocculence will be found on the lining membrane of the *upper eyelids*, although the ball of the eye be free, with sometimes a thickening, approaching to what is called granulation, about the inner angle of the eyelid. This thickening of the upper, is quickly followed by a villous and prominent state of the under lids. The sensation of sand becomes unpleasant, with a corresponding increase of lachrymal discharge, which, from its corrosive acrimony, produces much pain and irritation. The meibomean glands now enlarge, and pour out a proportionate discharge —limpid serum flows from all parts of the eye and eyelids, excoriating the parts over which it passes—the lower eyelids become tumefied and hard, from the degree of which, a prognosis may be formed respecting the severity of the disease. So great is the increase of secretion in this form of ophthalmia, that all the surrounding structures are, as it were, injected—and the chambers of the eye are so over-replenished with fluid, in many instances, as to cause a defect in vision.

3. The *third* form is the *purulent* ophthalmia. It is perhaps not more formidable, in reality, than the second form, as, in our author's practice, it has yielded as soon, and as certainly to remedy, as the complicated cases of the other variety.

"The eye becomes itchy; and the patient cannot be persuaded but that sand or dirt, as he expresses it, is lodged between the eyelid and ball of the eye. He endeavours to remove the disagreeable sensation by rubbing; which, not producing the expected relief, is immediately succeeded by obtuse pain, augmentation of the sensation of heaviness, and by a flow of fluid resembling tears, which, from being thin and watery at first, is converted into a puriform matter, wholly divested of the acrimony attending the serous discharge in the second form. If the eyelids be now examined, a thickening of the lining membrane of the upper is observable, and the lower are villous. As the puriform discharge augments, it becomes dense and tenacious: the capillary vessels on the ball of the eye and neighbouring parts, are in a state of irritation. This is considerably augmented by rubbing the ball, and the propensity to rubbing is irresistible. The vessels which nourish the conjunctiva and lining membranes pour forth a matter of a puriform nature, whilst those of the eye-lids internally, and also of the internal parts of the eye, secrete and discharge a quantity of whey-coloured serum, producing a tumefied state of the eyelids, and of the ball of the eye, which characterize the disease, and occasion the partial or total loss of vision. (The loss of vision is attributable to the existence of a turbid fluid in the aqueous and vitreous humours, the obtuse pain to the distention of the ball of the eye from extravasated fluid, and not to inflammation.) As the turgescence and prominence of the eyelids, which assume a bluish hue, increase, the vessels which nourish the conjunctiva and sclerotica augment in size; the texture of the former, being reticular and spongy, is injected with red fluid, not blood, or with pure and limpid serum, which, as the determination of fluid to the part is considerable, produces extraordinary distention of the conjunctiva. This is sometimes so great as to cause the distended mass to encroach on the cornea; and as the size of the latter appears diminished, to one-half probably, it excites the apprehension of such as are unacquainted with this form of the disease. To this state of the conjunctiva, oculists have given the name of chemosis." 10.

The disease invariably appeared, in the 64th regiment, in one or other of these three forms. The *first*, although simple in the outset, occasionally assumed an alarming character. The *second* variety had different modes of attack—was treacherous in the extreme—and liable to relapses under conditions apparently prosperous. The *third* (purulent) variety, generally observed a regular course—the discharge was removed within a short time from the date of admission, by remedial means, and convalescence was generally rapid and certain. Still, our author considers all these varieties as only modifications of one and the same disease—the discharges being merely symptomatic of the different degrees of the malady. The same plan of treatment was extended to all the three forms, " with a success unequalled in Southern latitudes."

*Methodus Medendi.* Our author considers ophthalmia, in every form, to be a local disease, dependent for its production on general causes, which augment the vascular action and secretions of the eye and neighbouring parts, in a similar manner as the secretions of the nasal cavity, &c. are increased during the prevalence of catarrhal affections, to which, he thinks, ophthalmia bears considerable analogy.

" The plan of cure adopted and pursued was as follows:—the patient, on admission, was confined to bed in a large and well ventilated ward, from which light was not excluded. (I conceive that the exclusion of air and light, as is the custom in the treatment of this disease, is often attended with lamentable consequences, always with injury.) He was placed on the spoon scale of diet, purged with calomel and colocynth, and afterwards with salts. The state of the eye-lids, whether the disease was of the purulent kind or not, was attended to. The upper eye-lids were examined; and whatever the appearance might be, whether villous, thickened, &c. blue stone in substance was rubbed over the surface, for a longer or shorter time, according to the condition of the membrane. If the eye-lids were thickened, as was often the case, or in a state which oculists call ' granulated,' as occurred sometimes, the passing of the blue stone over the surface was continued for a considerable time: if the appearance was only villous, a superficial and short application was sufficient. The application of blue stone to the surface of the everted eye-lid is attended with acute pain, and on one occasion has produced syncope; but the benefit which resulted, and which results in a short time, is far superior to what has been derived from any remedy hitherto recommended for the cure of this form of disease. Persons who are acquainted with the effects of blue stone on ulcers, &c. will probably revolt at the idea of touching the eye with a substance so irritating; but when they consider that its use has not in any one case been attended with danger, during an extensive application of it in the treatment of this disease; that, on the contrary, it has been uniformly followed by benefit; and that the discharge, whether purulent or otherwise, has been changed in its nature, suspended or suppressed in a few hours; the irritability, pain, and disagreeable sensations mitigated or removed, soon after the application, they cannot well refuse to give it a trial. The author confidently affirms it to have, in his hands, proved a remedy greatly superior to any other hitherto employed. In this case he speaks with confidence, as justified in his opinion by experience, on an extensive scale, in the acute as well as the chronic stages of the malady. It is comparatively safe and little troublesome, and it is seldom followed by ulcers or other bad consequences. Let the application of blue stone to the upper eye-lids, and not unfrequently to the lower, be followed by fomentations, repeated four or five times in the twenty-four hours. If vision had been impaired, an alteration for the better in that respect will generally be observed on the second day, the discharge, if great and attended with nictitation, will be changed in quality and much diminished in quantity: the heaviness and pain will be lessened, if not entirely removed, in so much, that the patient proclaims himself in a state of comparative happiness. A change for the better having been thus effected in a few hours, and danger in such instances obviated, it will be well, if the application of the remedy had been only superficial and transient on the first day, to repeat it on the second; but if it had been severe, and if the slough adheres to the lining membrane, as often happens where there is great debility of the parts from over distention of the vessels, it will be well to instil a solution of lunar caustic into the eye in the morning, in the proportion of ten grains to an ounce of water, and to repeat the fomentations as on the preceding day. A solution of lunar caustic, of the above strength, is an excellent remedy in this disease. It may be used at all periods, and, next to the blue stone, claims a preference to all others. Its action, when resorted to at an early period, tends to change and lessen the discharge, and to remove the pain and irritability, without causing any of those unpleasant symptoms which some have attributed to its use. It is certainly a remedy of great value, and may, in slight cases, or where the patient is averse from the application of blue stone, be depended upon as efficient means of cure. As an auxiliary in a severe or protracted disease, it ought never to be omitted. I have made trial of lunar caustic solution in different degrees of strength, viz. from one grain to half a drachm, dissolved in an ounce of water; and, after witnessing the effects of all, I gave the decided preference to the ten grain mixture. On the second day I usually give a purge of the magnes. sulph. and occasionally repeat the lunar caustic drops in the evening, or touch the lower eye-lids slightly with blue stone; after which fomentations are again employed. On the third day the blue stone or caustic drops are repeated, and fomentations applied to the eyes. Under this plan of treatment, varied according to circumstances, more than eighty of one hundred will recover, without the use of other means; and it may be presumed that, in consequence of the attention which is paid to the upper eye-lids, the condition which is commonly, but improperly, called "granular," will be prevented; relapses, although frequent, will be comparatively fewer than where the antiphlogistic plan is pursued; and ulcers, so common where copious bleeding is employed, will seldom appear; and when they do appear, they are of a mild kind, and rarely imply much loss of substance." 15.

Thus, the sulphate of copper, solutions of lunar caustic, purgatives, and fomentations, constituted the treatment of this disease, not only at its beginning, but throughout its whole course. Our author avers that abstraction of blood will rarely be necessary in this complaint, if the plan recommended be strictly attended to. Whenever deep-seated and acute pain, however, denoted internal disease (which was not often the case) recourse was had to local abstraction of blood by means of leeches, after which, the parts were carefully fomented, generally with relief. Dr. O'Halloran is thoroughly persuaded that ophthalmia, in its native or characteristic form, is a disease of less danger (as regards the safety of the eye) than is generally supposed—"provided the patient be placed in a *healthy situation, to which air and light have free access*." The loss of vision, he thinks, is owing, in nine cases out of ten, either to inflammation produced by over-distention of the eyeball from extravasated fluid—to over-depletion by the lancet—or to ill treatment in confined apartments, where the air becomes contaminated.

2. *Chronic Ophthalmia.* It is extremely difficult in this, as in all other inflammations, to mark the limit between the acute and chronic stages. And yet it is a matter of importance to be able to ascertain the commencement of the chronic form of ophthalmia, as it may be necessary to alter the plan of cure—to remove the patient from bed into the pure air, &c. We must pass over Dr. O'Halloran's speculations, physiological and pathological, on the state of the arteries and veins in acute and chronic ophthalmia. We do not see that they add any thing towards the elucidation of the proximate cause of the disease. He acknowledges indeed that the main part of the diagnosis must be founded on the effect of medicines. We shall give this diagnosis between acute and chronic ophthalmia in Dr. O'Halloran's own words.

"If we apply caustic or bluestone to the upper or under eye-lids in the chronic ophthalmia so as to produce a slough, the vessels, being destitute of power, are unable to throw it off in less than from three to six days; whilst in the acute stage, where the arteries are possessed of high action, the same quantity of slough is ordinarily removed in one night. This is a fact of practical importance. It furnishes us with the means of ascertaining whether the case before us be of the acute or chronic character; and no injury can result from the experiment, for bluestone is equally serviceable in both stages; in addition to this, we also find, that when the disease assumes the chronic form, the veins on the upper eyelids, forehead, and temples are gorged with blue blood, and as they appear wholly destitute of activity, the blood which they contain is in a state of demi-coagulation; the arteries also of the ball of the eye are inordinately distended and hard to the touch; they appear destitute of the p wers of propulsion." 27.

The next pathological discussion is on the *granulations* which obtain, or are said to obtain, in the eyelids of ophthalmic patients. These granulations, our author avers, exist only in the imaginations of oculists. Similar appearances are found in the eyes of those who never had ophthalmia. But as this is a point of some importance, we shall let Dr. O'Halloran speak for himself.

"In illustration of the absurdity of the supposition of the existence of granulations in the eye-lids of ophthalmic patients, I must take the liberty of observing, that, in the first place, we see in the eye-lids of persons who never suffered from the disease an appearance similar to what is commonly designated 'granular.' This may be proved to the satisfaction of the most prejudiced, by the examination of the eye-lids of any body of men, viz. a military corps or regiment. In every large body of men, persons are to be found, whose eye-lids are overspread by villous flocculencies or fungous productions analogous to what has been denominated 'granulations,' notwithstanding that from youth they may have enjoyed health, or absolute immunity from the affection under notice.

"Secondly, We not unfrequently observe, that a thickening of the lining membrane of the upper eye-lids, similar to what is called "granular," is noticeable as soon as the disease manifests itself on the eye; a fact that places in a ridiculous light this granular doctrine, which has obtained the support of some of our most respectable oculists.

"Thirdly, We find that the state of the eye-lids under notice exists in all varieties of ophthalmia, simple or complicated, watery or purulent; and as granulations, if we reason from analogy, are only produced in other parts of the human frame where pus exists, we cannot but be astonished at their appearance on the eye-lids where pus had not been seen.

"Fourthly, We find that by the plan of cure which I have recommended, the purulent discharge, however copious, is arrested in a few days; probably in a few hours; and that the disease runs its subsequent course without the production of pus; yet the state of the eyelids called 'granular' exists whenever the cure is protracted, and it even occasionally exists in defiance of our best means of counteraction.

"Fifthly, It appears contradictory to suppose, that granulations can take place without a breach of surface or loss of substance.

"Finally, There is a considerable variety in that state of the lids called 'granular;' sometimes an appearance similar to the finest sand-paper, rough, hard, &c. presents itself; at other times a part of the eye-lid is covered with excrescences, whilst the remainder is smooth and villous. Again, we notice a great diversity in the size and elevation of those substances; the whole eye-lid is in many cases covered with them, intermixed occasionally with fatty tumours. Those appearances do not obtain in other diseases. The granulations of ulcers may vary in size and number, but they never exhibit the variety which is seen in this disease.

"From a consideration of the foregoing circumstances, I think I am warranted in concluding that granulations do not exist in this disease; that the appearances which have given origin to the conjecture (for it is nothing more), are simply enlargements of a fungous nature from the glands and vessels, the former, if I mistake not, being far more numerous than has been supposed; that similar enlargements take place in scrofulous habits where the glands in other parts are diseased; that, in such cases, the affection of the glands of the eye-lids is sometimes the cause and not the effect, of what is called inflammation; that the vessels which are numerous, when they intersect one another in a state of distention, become united and in part obliterated, not only from the effects of the union, a stagnant state of the circulation perhaps, but from the pressure of the glands in an enlarged state; and that the whole united, and deficient in excitement, is formed into an irregular mass, which, in many instances, acquires a degree of firmness unknown in granulated surfaces." 30.

*Treatment of Chronic Ophthalmia.* In military practice this is a troublesome and sometimes very obstinate disease—probably from the circumstance of soldiers applying irritating substances to their eyes, for the purpose of evading duty. The sudden transitions from a favourable to an unfavourable state, could not be otherwise accounted for. "The women, children, and men of good conduct, rarely experienced such changes."

Whenever the disease of the eyelids resisted the means of cure already described—or, in other words, assumed a protracted course, the upper eyelids became thickened and diseased. As long as this state continued, so long were the marks of inflammation observable on the ball of the eye and inferior eyelids.

"For the cure of the thickened state of the eye-lids, commonly denominated 'granular,' it will be necessary to apply bluestone in substance daily, provided the irritation produced by it is of temporary duration; but should it occasion unusual pain, *which rarely happens*, it will be proper to instil, on the following day, a solution of lunar caustic into the eye, of the strength of ten grains to an ounce of water, and to foment three or four times in the twenty-four hours. Where bluestone disagrees (which is seldom the case) and the fomentations are unavailing in allaying irritation, or the lunar caustic drops possess but little efficacy against the disease of the eye-lids, recourse may be had to lunar caustic in substance, which, when slightly rubbed over the internal surface of the upper or lower eye-lids, frequently removes the irritation, and sometimes even cures the disease. The caustic in substance proved injurious occasionally; and, in one instance, opacity of the cornea followed its use when applied in force for the removal of the disease of the upper eyelid. It ought not to be employed for that purpose; for when used to an extent sufficient to produce a deep slough, it generally does harm. Its utility, therefore, in chronic ophthalmia consists in diminishing irritability, and, for that end, the slighter the application the better. It is not, however, good reason to abandon the use of a remedy, because injury is sometimes produced by it. We are warranted at trying different agents, and the daily use of remedial means is admissible, except in cases where extraordinary irritation exists. If after repeated trials they all fail of mitigating pain and allaying irritation, a respite of two or three days will be advisable, merely keeping the parts clean with warm or cold water. I think that I have saved eyes by the use of the caustic where it previously had done harm. As the irritation subsided, the employment of the caustic was attended with benefit; and the cure in all probability was effected by that remedy alone, according to the modified manner of management." 34.

But the most efficacious remedies will sometimes fail. Many causes, constitutional as well as atmospherical, occasionally interpose, so as to counteract the effect of remedial applications. The practitioner must bear in mind that, in certain habits of body, TIME, and an attention to constitutional derangements, with change of air or climate, will effect what medicine failed to accomplish. The preventive measures against relapse consist in the occasional application of blue-stone to the eyelids during the period of convalescence.

"It is astonishing how quickly the state of the eye-lids, denominated 'granular,' is removed by the bluestone, if the subject be not of scrofulous habit, or the eye-lids spongy, watery, or covered with fatty dry lumps. In the latter case, the knife will be necessary for the removal of the tumours; after which a solution of bluestone will complete the cure. *The case in question is the only one in which, in my opinion, the knife is admissible to the internal surface of the eye-lids.*" 38.

So far as Dr. O'Halloran's observations extend, fomentations with warm water are always beneficial, but washes with alumen and zinc, are useless if not prejudicial. For some criticisms on the work of Mr. Travers, we must refer the reader to the original.

*Ulcers, and Lymphous Effusion.* Ulcers are liable to occur in all stages and forms of ophthalmia—but they are more dangerous and prevalent in the chronic than in the acute stage; "they seem to derive their origin from two causes, viz. excess of action, and deficient circulation." In the *former* case, these ulcers do not alone affect the conjunctiva, but even portions of the cornea, if remedies be not seasonably applied. In the *second* description of ulcer, portions of cornea which had been previously nourished by blood, but which are now deprived of it, disappear by absorption, without pain, irritation, or loss of the covering portion of the conjunctiva. The ulcer in this case is clean and healthy in appearance, and not surrounded by undue vascularity. This last description of ulcer was rare in the 64th Regt. and was difficult of management, when it did occur.

When an ophthalmic patient, observes our author, whose eye is ulcerated, is confined in impure or stagnant air, it is impossible to remedy the evil by treatment. Medicine is of little or no avail; but removal to a pure atmosphere has a sudden and striking effect upon the character of the ulcer. The patient, in our author's opinion, should not, after the third or fourth day be allowed to wear a veil over his eyes, and ventilation should be free. These ulcers are remediable by the means already pointed out.

"They seldom or never appear, except where the upper eye-lids are implicated; and, whatever might have been the duration of the disease, in addition to the slight application of caustic to the ulcer itself, I conceived that the use of blue-stone to the eyelids was necessary; and I have the satisfaction to say, that the good effect was generally evident in less than twenty-four hours. On the next day, whatever might be the state of the ulcer, I dropped a strong solution of caustic into the eye, and repeated it in the evening. Under the alternate use of the sulph: cup: and solut: nit: argent: (viz. ten or twenty grains to the ounce of water,) if the air of the ward was pure, the diet proper, and the state of the bowels well regulated, the ulcers generally healed in a given time. If, however, the ulcers do not yield to the remedies recommended, but continue obstinate after the disease is removed from the eyelids, the eye itself, as influenced by the ulcer, being watery, red, and irritable, I apply the lunar caustic in substance lightly, either to the lower or upper lids; and this application, although sometimes hurtful when extensively used in cases of constitutional irritability, is generally useful by removing irritation, and inducing a favourable change in the condition of the sore." 51.

*Lymphous Effusion.* This was a common occurrence in protracted cases—rarely appearing in the earlier periods. Whenever unusual irritation took place suddenly in advanced stages of the disease, effusion of a lymphous character was generally observed at the superior margin of the cornea. This effusion, our author often suspected to be the product of artificial irritation applied by the patient himself. It takes place from a cluster of vessels which discharge a dusky-coloured jelly-like substance that descends to a certain point to which it seems to adhere. It seems to destroy the bond of union between the conjunctiva and external margin of the cornea. "When the newly-formed parts become, as it were, organized, the irritation, which is great during their formation, subsides, and the removal of the whole is afterwards effected by the combined efforts of nature, time, and artificial remedies." 55. When lymph is thrown out in the manner described, it is difficult to arrest its progress. Topical bleedings, solutions of lunar caustic, and fomentations failed. In some later trials Dr. O'Halloran applied the lunar caustic in substance on the effused lymph, and also to the distended trunks of the vessels which poured it out. By this means the disease was arrested. He has also divided the conjunctiva over the trunks of the vessels and applied caustic to them, having previously syringed the eye for the purpose of removing the blood which the division occasioned. This plan answered where the eyes were large, but in small eyes it was found impossible to expose the vessels so as to be operated on in the manner described.

We have now finished the ophthalmic portion of Dr. O'Halloran's work, and it is evident that if the practice here delineated prove successful in the hands of others, a great advance will have been made in this branch of surgery. It is gratifying to be able to state, that a nearly similar practice has, for some time, been carried on at Chatham, under the direction of Mr. Melin, who superintends the Ophthalmic Institution of the army there, and with far greater success than attended the former depletory measures.

The portion of Dr. O'Halloran's volume, embracing the subject of remittent fever, must come under review at another time and place. We part from this zealous and intelligent officer, in the mean time, with sentiments of much esteem and respect.

